# Enhancing the Antifungal Activity of Griseofulvin by Incorporation a Green Biopolymer-Based Nanocomposite

**DOI:** 10.3390/polym13040542

**Published:** 2021-02-12

**Authors:** Amr Shehabeldine, Hany El-Hamshary, Mohamed Hasanin, Ayman El-Faham, Mosaed Al-Sahly

**Affiliations:** 1 Botany and Microbiology Department, Faculty of Science (Boys), Al-Azhar University, Cairo 11884, Egypt; dramrshehab@azhar.edu.eg; 2 Chemistry Department, College of Science, King Saud University, P.O. Box 2455, Riyadh 11451, Saudi Arabia; aelfaham@ksu.edu.sa (A.E.-F.); 433107098@student.ksu.edu.sa (M.A.-S.); 3 Department of Chemistry, Faculty of Science, Tanta University, Tanta 31527, Egypt; 4 Cellulose and Paper Department, National Research Centre, Dokki, Cairo 12622, Egypt; 5 Department of Chemistry, Faculty of Science, Alexandria University, P.O. Box 426, Ibrahimia, Alexandria 21321, Egypt

**Keywords:** biofilm, Griseofulvin, confocal laser scanning microscopy, *Candida* spp., polysaccharides

## Abstract

Fungal biofilms have caused several medical problems, resulting in significant morbidity and mortality as well as poor response to antifungal drugs. The current study was designed to evaluate the enhancement of antifungal and anti-biofilm activity of Griseofulvin-loaded green nanocomposite-based biopolymers (Ge-Nco) of glycogen and gelatin against different strains of pathogenic *Candida* species. The prepared Ge-Nco was characterized using Fourier-transform infrared (FT-IR), X-ray diffraction pattern (XRD), scanning electron microscopy-energy dispersive X-ray (SEM-EDX) and transmission electron microscope (TEM). In addition, the morphology of the mature biofilm and the inhibition of biofilm was monitored and visualized using confocal laser scanning microscopy (CLSM). The minimal inhibitory concentrations (MIC) and (IC50) of Griseofulvin alone and the prepared Ge-Nco against three different strains of *Candida* sp. were determined according to Clinical and Laboratory Standards Institute (CLSI) method. The effects of Griseofulvin alone and Ge-Nco on the tested *Candida* sp. biofilm formation were determined by the crystal-violet staining protocol. The biofilm inhibition potential of Ge-Nco against the tested *Candida* sp. was detected and depicted under CLSM (2.5 D view). The findings depicted that Ge-Nco was prepared in nanometer size (10–23 nm). The observed minimum inhibitory concentration (MIC) of Griseofulvin alone and Ge-Nco against three different *Candida* sp. were found to be in range 49.9–99.8 μg/mL and 6.24–12.48 μg/mL, respectively. These results provide evidence for implementing efficient antivirulence approaches against three different *Candida* sp. that would be less likely to foster the emergence of resistance.

## 1. Introduction

There have been recent advances in eco-friendly and cost-effective natural and synthetic materials as therapeutic agents. *Candida* species are extremely dangerous infectious agents to humans. They are liable for 90%–100% of mucosal pathogens and the fourth leading cause of nosocomial infections attributing a 35%–50% mortality and morbidity to patients and critical individuals with immunodeficiency. There has also been a marked rise in the occurrence of fungal human infections over the past 40 years [1]. Infections of this type can either be superficial, impacting the skin, hair, nails and mucosal membranes, or chronic, involving large organs of the body [2]. Many factors have been reported relating to this increased incidence of fungal disease, many factors have been reported, but it is widely agreed that perhaps the enhanced and extensive use of such medical methods is required. Thus, immunosuppressive treatments, invasive diagnostic interventions and, the use of broad-spectrum antibiotics, are critical [3].

Microbial groups of surface-attached cells enveloped in a self-produced matrix of extracellular polymeric substances can form biofilms [4], and they are produced in response to several environmental and physical indicators, such as high cell density, nutrient deficiency, and physical pressures, they are produced [5].

Scientists are increasingly understanding the importance of studying the biofilm species rather than planktonic models because of their therapeutic relevance when describing the pathogenic propensity of microorganisms [6]. Emerging technologies are expected to create newer and safer antifungal agents with broad-spectrum action that inhibits and destroys the virulence factors involved in the pathogenicity of life-threatening *Candida* sp. due to the production of many different extracellular hydrolytic tissue-damaging enzymes, hyphae transfer, and the development of highly drug-resistant biofilm in *Candida* sp. isolates [7].

In immunosuppressed conditions, fungal infections caused by *Candida* species have been one of the primary causes of mortality and morbidity, and, candidemia treatment remains a daunting great challenge to the widespread emergence of antifungal drug resistance to be used in clinical practice [8]. For several years, the majority of reports of candidemia have been triggered by *Candida albicans*, and there is urgency to find alternate antifungal agents.

Nanomaterial-based strategies have received substantial attention from the research community because of their unique antimicrobial potency as a consequence of the combination of their small size and high surface-to-volume ratio, which enables interaction with microbial membranes. Because of their stability and future biotechnological purposes, inorganic antibacterial agents, including metallic and metal oxide nanoparticles (NPs) are more beneficial than organic compounds. Biopolymers are a broad group of natural polymers, which are defined as natural source polymers [9,10,11]. The use of biopolymers in pharmaceutics can improve drug performance and increase the drug’s half-life [12,13]. Glycogen is a natural polysaccharide usually produced in animals as energy storage [14]. Additionally, gelatin is one of the animal source biopolymers used widely in many pharmaceuticals [15]. Glycogen and gelatin have a high safety profile, and are biocompatible and biodegradable biopolymers with no toxicity. Therefore, in this study, we prepared bio-based Ge-Nco-loaded Griseofulvin and characterized it at the nanoscale to increase the efficiency of antifungal and anti-biofilm activities against three clinical strains of *Candida* spp.

## 2. Material and Methods

### 2.1. Materials

Glycogen was purchased from Schuchardt Munchen, Germany. Gelatin type-B from bovine skin was obtained from Sigma-Aldrich, Steinheim, Germany. Griseofulvin was purchased from Sigma-Aldrich, Steinheim, Germany. Sabouraud’s Dextrose Agar (SDA) and Sabouraud’s Dextrose Broth (SDB) were purchased from Himedia (Delhi, India). Live/Dead^®^BacLight™Bacterial Viability Kits were purchased from Thermo Fisher Scientific, (Waltham, MA, USA). Additionally, all other reagents were of analytical grade and used as received.

### 2.2. Preparation

Typically, one gram of each glycogen and gelatin were separately dissolved in 100 mL of Millipore water. After complete dissolution, 100 mL of each solution were mixed in 500 mL round flasks under continuous stirring at 1500 rpm for 3 h, keeping in mind that the stirring temperature was raised to 70 °C. At the end of stirring, the stirred solution was left to cool down to the ambient temperature. Griseofulvin (1 mg/mL) was dissolved in Millipore water under magnetic stirring [16]. Then, 10 mL of the dissolved Griseofulvin solution was added drop by drop to the glycogen–gelatin admixture solution with contentious stirring at 1500 rpm for 3 h at 40 °C. The prepared Ge-Nco was lyophilized at −80 °C and kept in a refrigerator for further characterization.

### 2.3. Characterization

The newly functional groups for the prepared samples were monitored using Fourier-transformed infrared (FT-IR) spectroscopy (Spectrum Two IR Spectrometer from PerkinElmer, Inc., Shelton, CT, USA). Using KBr, 0.002 g per sample was completed to 0.2 g. All spectroscopy was performed with 32 scans and 4 cm^−1^ resolutions in wave numbers ranging from 4000 to 450 cm^−1^. X-ray diffraction (XRD, Shimadzu 7000, Kyoto, Japan) was used to assess the crystal structure of the characterized samples. A Quanta 250 FEG (field emission gun) scanning electron microscope (SEM) was used with energy dispersive X-ray (EDX) analysis to detect the surface morphology of the prepared Ge-Nco and its native materials, which were mapped with an accelerating voltage of 30 KV. A transmission electron microscope (TEM, Model JEM2010, Kyoto, Japan) was used to examine the particle size and morphology of the prepared products.

### 2.4. Influence of Minimum Inhibitory Concentration on Germination and Inhibitory Concentration of Compounds Required for 50% Inhibition of Germinated Candida Strains

The minimum inhibitory concentration (MIC) of Griseofulvin compound and Griseofulvin loaded in green nanocomposite-based biopolymers (Ge-Nco) against three different pathogenic *Candida* species: *C. parapsilosis* ATCC 90018, *C. tropicalis* ATCC 750 and *C. albicans* ATCC 90028 was determined using the reference method of the Clinical and Laboratory Standards Institute (CLSL) for broth dilution antifungal susceptibility testing with some modifications [17]. The MIC of the prepared Ge-Nco was determined using the two-fold serial dilution method, and the final concentration of Ge-Nco ranged from 100 to 3.12 µg/mL of amphotericin B dissolved in DMSO (5 μg/mL), which was used as a positive control. The MIC was recorded as the lowest concentration that produced complete suppression of visible growth while IC50 was the concentration of compounds required for 50% inhibition of germinated *Candida* strains. Triplicate samples were used for each test, and MIC and IC50 values were calculated. Additionally, the growth percentage was calculated using Equation (1). Death kinetic curves were also plotted, by controlling the *Candida* cells through estimated IC50 values of Griseofulvin and prepared Ge-Nco [18].

(1) $$Growth\%\;=\left(\frac{OD_{410}\;\mathrm{of}\;\mathrm{wells}\;\mathrm{containing}\;\mathrm{nanocomposite}}{OD_{410}\;\mathrm{of}\;\mathrm{nanocomposite}\;\mathrm{free}\;\mathrm{wells}\;}\right)\;\times \;100$$

### 2.5. Evaluation of Anti-Biofilm Activity

*C. albicans* biofilms were developed using a static model following a previously published protocol [19]. Briefly, overnight cultured *Candida* cells were suspended with an OD600 of 0.1 in PDA medium containing Ge-Nco at a sub-inhibitory concentration for each strain. A measure of 100 μL of the suspension was then added into 96-well microtiter plates. The plates were covered with lids and incubated at 37 °C. After 24 h of incubation, the plates were washed with PBS, and biofilm activity was detected using the crystal violet staining protocol [20]. After discarding the liquid mixture, the wells were washed with sterile water twice and stained with 0.1 mL 0.4% crystal violet for 15 min. Then, the samples were rinsed twice with distilled sterilized water and the dye bound to the biofilm was solubilized by adding 95% ethanol. Absorbance of the isolated dye was measured quantitatively at 540 nm. The biofilm inhibition percentage was calculated using the following formula [21]:

(2) $$Biofilm\;Inhibition\%\;=\left(\frac{OD\;\mathrm{growth}\;\mathrm{control}-\mathrm{OD}\;\mathrm{sample}}{OD\;\mathrm{ogrowth}\;\mathrm{control}\;}\right)\;\times \;100$$

### 2.6. Visualization of Biofilm Inhibition by Confocal Laser Scanning Microscopy (CLSM)

The suspension of the *Candida* strains was diluted with solutions of both 0.9% saline and 0.5 McFarland standard. After that, 1 mL of cell suspension was dispensed with a submerged sterile glass microscope (13 mm in diameter and 0.2 mm thick) containing a Ge-Nco compound at 0.5 × MIC and 0.25 MIC into the wells of microtiter plates. Plates were continuously incubated for 48 h at 28 °C. The covers were carefully cleaned using 0.01 M PBS to eliminate the non-adhered *Candida* cells and colored with 500 μL of SYTO 9 (gray fluorescent with viable cells) and propidium iodide (red green fluorescent with dead cells) mixed dye solution after the incubation period. The Live/Dead^®^ BacLightTM Bacterial Viability Kits were used for 15 min in dark and ambient conditions [22]. Subsequently, a ZEISS LSM 710 confocal laser scanning microscope (CLSM; Carl Zeiss, Jena, Germany) was used for imaging [23]. Using ZEN 2.3 software, image processing and eventual editing were performed. The prepared biofilm was utilized as the control sample.

### 2.7. Statistical Analysis

All the tests were performed in triplicate, the outcomes were expressed as a means ± standard deviation.

The data were analyzed by using SPSS version 17.0. A two-sided P-value of less than or equal to 0.05 was considered significant.

## 3. Result and Discussion

### 3.1. Characterization

#### 3.1.1. FT-IR

**Figure 1:**
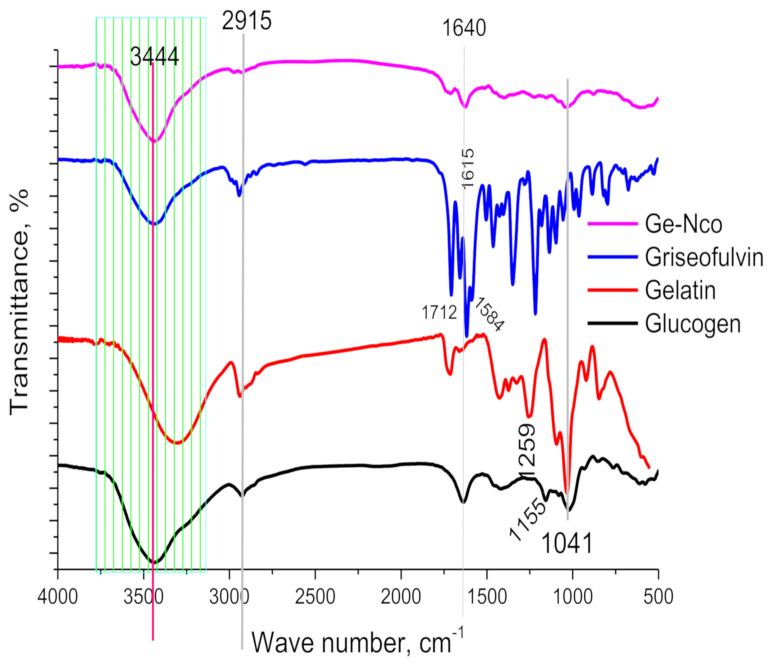
Fourier-transformed infrared spectroscopy of green nanocomposite-based biopolymers and their raw materials.

The prepared samples were examined using FT-IR, XRD, SEM, EDX and TEM to elucidate their structure. The FT-IR spectra of nanocomposite materials and Ge-Nco are presented in Figure 1. Characteristic peaks were observed for glycogen at 3397, 2915, 1640, 155, and 1038 cm^−1^, indicating O–H stretching, C–O stretching, the over tone of the hydroxyl group, and stretching of the Carbon/Oxygen single bond and CH_2_–O–CH_2_, respectively [24]. Additionally, the pure gelatin showed characteristic peaks at 3291, 2931, 1719, 1259 and 1041 cm^−1^, which were attributed to overlapping of NH and OH bond stretching vibration, carboxylic group stretching vibrations, C=O stretching, NH bending vibration of the amide group and C–O–C of the amide bond in gelatin stretching vibrations, respectively [25]. In context, the pure Griseofulvin was recorded a clear peaks characteristic the Griseofulvin at 3436, 2944, 1712, 1615 and 1584 cm^−1^ indicating adsorbed water vapor from the environment, vibrations in CH_2_ groups, the C=O stretch of the benzofuranone ring, cyclohexanone carbonyl hydrogen bonds and C=C stretch of the cyclic ring, respectively [26,27,28]. However, Ge-Nco had a lower peak at 500–1700 cm^−1^ than the pure drug. Moreover, the characteristics peaks of the pure drug at 1715 and 1625 cm^−1^ were reduced to small peaks with the shift to a higher frequency. Additionally, the peak at 1584 cm^−1^ in the pure drug disappeared in Ge-Nco. These results confirm that the drug was included into the biopolymer network and distributed according to the 3D structure of the biopolymer helix.

#### 3.1.2. Crystallography

**Figure 2:**
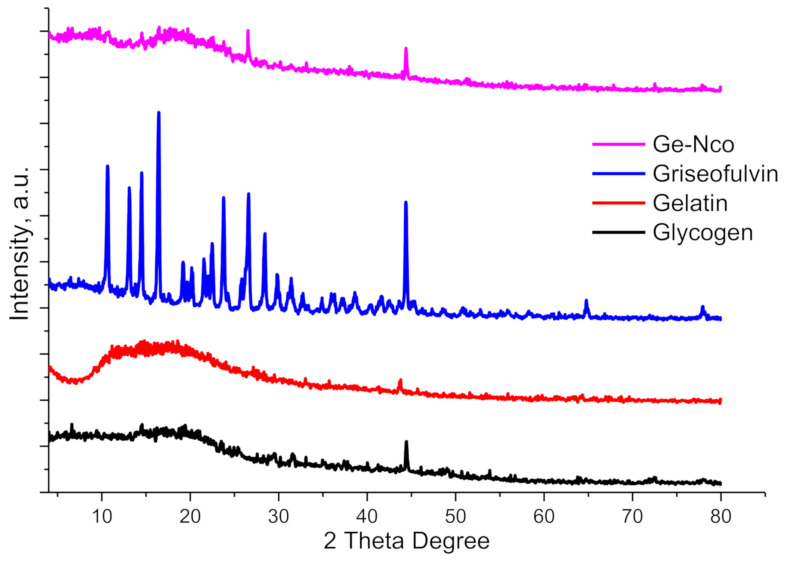
X-ray diffraction of the green nanocomposite-based biopolymers and their raw materials.

The crystallography of pure materials and prepared Ge-Nco were observed using XRD as showed in Figure 2. The pure glycogen diffraction was performed as a broad peak at 2θ~20° [29,30]. The gelatin pattern indicates pure gelatin with broad band close to glycogen pattern, indicating the amorphous structure that is characteristic of most natural and non-modified biopolymers [31,32]. Diffractograms of pure Griseofulvin clearly show that the drug is crystalline, as demonstrated by numerous sharp and intense peaks [33]. Additionally, the pattern shows significant characteristic peaks at 16.3, 26.65, 31.3, 32.7, 37.24, 38.69 and 44.4°, which corresponded to d-spacing at 5.4, 3.3, 2.8, 2.7, 2.4, 2.3 and 2.1, respectively [34,35]. In this context, the Ge-Nco was showing an amorphous region due to the biopolymers. However, the Ge-Nco observes a crystalline peak at 26.5, 38, 44.3. These findings are supported by the FT-IR results, and support the loading of Griseofulvin into a prepared composite.

#### 3.1.3. Topography Study

**Figure 3:**
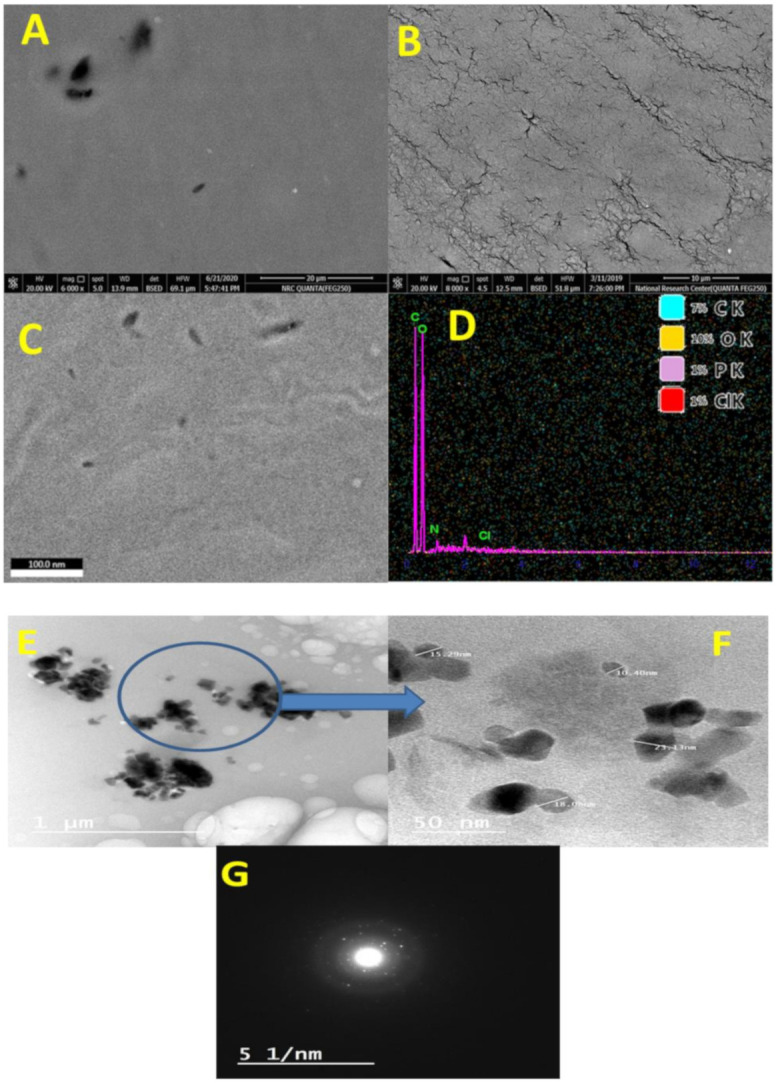
Scanning electron microscope topography of glycogen (**A**), gelatin (**B**), green nanocomposite-based biopolymers (Ge-Nco) (**C**), and mapping as well as energy dispersive X-ray chart (**D**). Transmission electron microscope of green nanocomposite-based biopolymers at low magnification (**E**), at high magnification (**F**), and diffraction of nanoparticle (**G**).

The surface and particle size performance of Ge-Nco were studied using SEM. The SEM topography of glycogen, gelatin, and Ge-Nco are presented in Figure 3A–D. Both biopolymers (glycogen Figure 3A and gelatin Figure 3B) had a conventional flat film shape without any unique surface characters. However, the Ge-Nco had a rough surface with the appearance of a new texture. Moreover, the high magnification SEM imaging (Figure 3C) indicated that the surface of the Ge-Nco is not flat and contains nanosized particles, which were studied by EDX & mapping (Figure 3D) and showed the presence of Cl atom due to the Griseofulvin chemical composition. Additionally, the TEM imaging of Ge-Nco in Figure 3E,G confirmed that the particle size of Ge-Nco was 10–23 nm. Additionally, diffraction of the Ge-Nco pattern emphasized that the particles are crystal. Overall, the topographical characterizations, as well as EDX and mapping emphasized that the Ge-Nco falls in the nanoscale range with adequate crystallinity, which indicates the loading of the drug.

### 3.2. Anticandidal Susceptibility Testing

The MICs of Griseofulvin and nanocomposite (Ge-Nco) against different strains of *Candida* spp. were determined using the turbidimetric antifungal assay. Serial dilution anti*candida*l assays revealed the hydride nature of synthesized Ge-Nco. The MIC and IC50 of prepared Ge-Nco were determined and are presented in Table 1. Depending on the *Candida* sp., the MIC of Griseofulvin and Ge-Nco were found to be in range 49.9–99.8 μg/mL and 6.24–12.48 μg/mL, respectively. The synthesized Ge-Nco has superior anti*candida*l activity against three tested *Candida* sp. when compared with Griseofulvin. This behavior could be due to the regulatory phenomena of biopolymers where the active ingredient dispersed over all the polymer helix and became more expressed [13]. It is evident from this experiment that Ge-Nco displayed less than twice the MIC and IC50 values from Griseofulvin in all tested *Candida* strains. A possible explanation for this is the controlled release of Griseofulvin from the composite cavity. The high risk of invasive *Candida* infections within the increasing population of immunocompromised patients, along with the emergence of resistance to the most common anti-*Candida* drugs for resistance *Candida* spp., also due to biofilm phenotypes, requires the development of new anti-*Candida* agents. In this context, nanotechnology could represent a very promising alternative to treat planktonic *Candida* and for developing biofilm-embedded *Candida* cells [36]. Moreover, the *C*. *tropicalis* strain exhibited higher MIC rates than the *C. albicans* and *C. parapsilosis*. These implied that these virulence phenotypes and antifungal resistance were collectively responsible for the prevalence shift of *C*. *tropicalis*) [37].

### 3.3. Inhibition of Fungal Biofilm by Green Nanocomposite-Based Biopolymers

**Figure 4:**
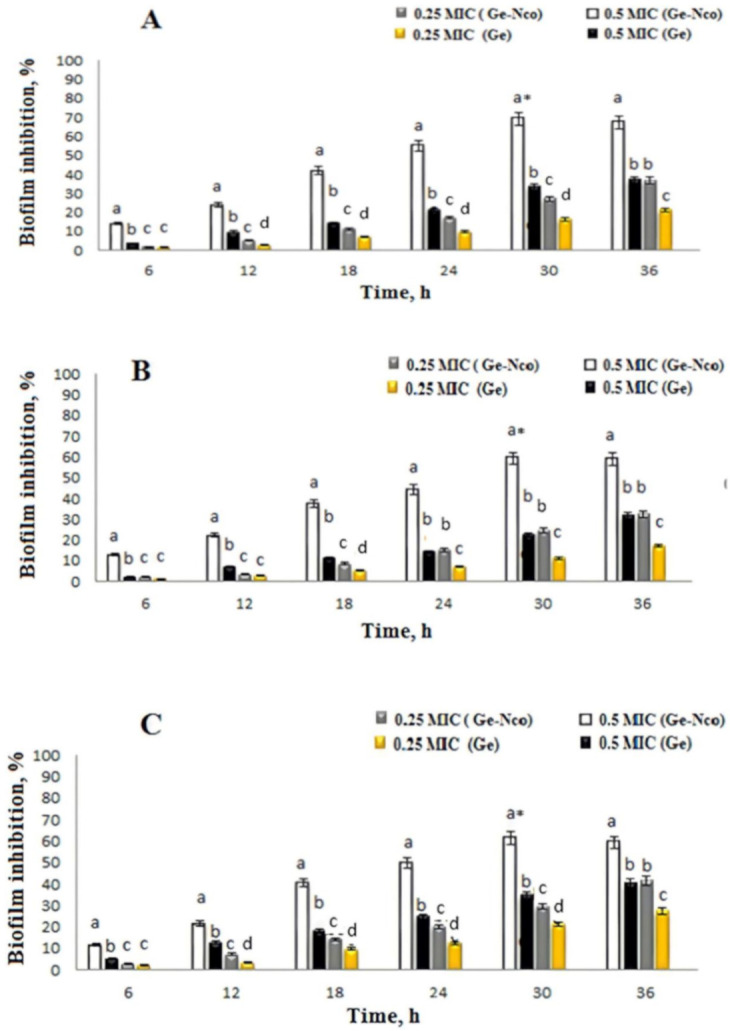
Different letters represent the significance between Inhibition of biofilm after the exposure of 0.5 MIC and 0.25 MIC concentrations of Ge and green nanocomposite-based biopolymers (Ge-Nco) on *Candida parapsilosis* (**A**), *C. tropicalis* (**B**), and *C. albicans* (**C**), respectively at each time of incubation. * denote the highest biofilm inhibition recorded after 30 h of incubation which significantly different (Holnm–Sidak’s test, BI = 68%, *p* = 0.001). a, b, c and d indicate the significant difference *p* < 0.05, *p* < 0.01, *p* < 0.005 and *p* < 0.001, respectively.

**Figure 5:**
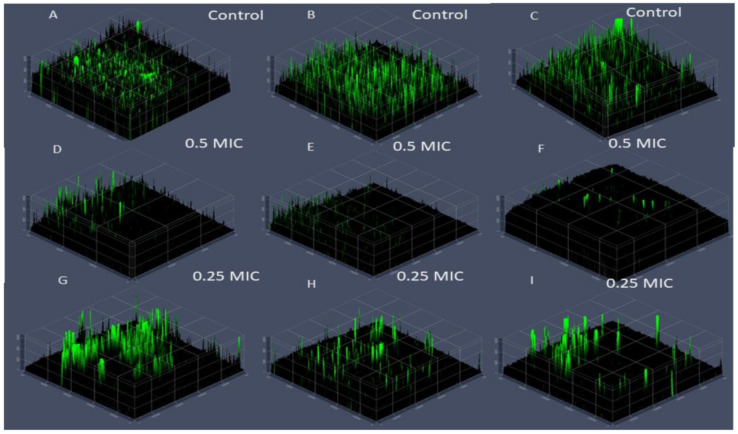
Anti-biofilm activity of green nanocomposite-based biopolymers against *Candida parapsilosis* (**A**,**D**,**G**), *C. tropicalis* (**B**,**E**,**H**) and *C. albicans* (**C**,**F**,**I**) through confocal laser scanning microscopy (2.5D view). Arrows indicate the thickness of *Candida* biofilms.

Using green synthesized nanocomposite modalities has been proven to have anti*candida*l activity and results were observed an excellent MIC and IC 50 values as presented above. Consequently, treat *Candida* biofilm-associated infections as well as biofilm virulence for drug resistance strains towered antifungal drugs were attracted substantial attention to evaluating for Ge-Nco [38]. The subinhibitory concentrations of Ge-Nco on biofilm inhibition effect were studied. The comparison of anti-biofilm activity showed that Ge-Nco performed as an anti-biofilm activity with statistically significant (*p* < 0.05) inhibition of biofilm formation of *C. parapsilosis* ATCC 90018, *C*. *tropicalis* ATCC 750, and *C. albicans* ATCC 90028 when compared with the control treatment (69.25%, 59.31%, and 61.5%, respectively, at 0.5 MIC) (Figure 4). However, a smaller effect was noted for Griseofulvin against *C. parapsilosis* ATCC 90018, *C. tropicalis* ATCC 750, and *C*. *albicans* ATCC 9002, with inhibitory rates 37.13%, 31.61%, and 40.23%, respectively at 0.5 MIC. The biofilm inhibition potential of Ge-Nco against the tested *Candida* species was depicted under a CLSM (2.5 D view) (Figure 5). The control slide showed well developed biofilm growth of *Candida* spp. However, the biofilm of *C. albicans*, which was treated with 0.5 MIC and 0.25 MIC showed reduced growth compared with the control treatment. Targeting a virulence factor, such as biofilm formation, is particularly attractive for developing antifungal resistance because it’s focused on potential targets in these eukaryotic cells [39]. 

The above results were emphasized that the Ge-Nco could prevent biofilm production without interfering with the cell viability at sub-MIC concentrations, where the experiments were performed using three clinical strains of *Candida* spp. with the high propensity and biofilm formation. Additionally, the cells from populations that evolved in the presence of Ge-Nco and Griseofulvin were enhanced biofilm-inhibitory activity compared with the effect of only Griseofulvin. Thus, we conclude that this antivirulence strategy is highly unlikely to foster the emergence of resistance.

## 4. Conclusions

The present study aimed to prepare effective green nanocomposite-based biopolymers namely, glycogen and gelatin loaded with Griseofulvin via a green method to enhancement anti*candida*l activity and reduce biofilm formation of *Candida* sp. The current study explicated that the inhibitory and antibiofilm activity of Ge-Nco against three tested *Candida* strains at 0.5MIC induced biofilm inhibition with an inhibitory rate 69.2%, is displaying much less than double times of MIC and IC50 values from Griseofulvin only in all tested *Candida* strains. Moreover, the further investigations revealed the Ge-Nco is abundantly acting as good antibiofilm. These results provide evidence for implementing efficient antivirulence approaches against three different *Candida* spp., namely *C. parapsilosis*, *C. tropicalis* and *C. albicans*, which would be less likely to foster the emergence of resistance.

## Figures and Tables

**Table 1:** Anti*candida*l susceptibility testing: estimation of the minimum inhibitory concentration (MIC) and the concentration of compounds required for 50% inhibition of germination (IC50) of all *Candida* strains.

	*C. parapsilosis*		*C. tropicalis*		*C. albicans*	
	MIC	IC50	MIC	IC50	MIC	IC50
Griseofulvin	49.9 ± 0.3	199.6 ± 0.3	99.8 ± 0.2	199.6 ± 0.2	49.92 ± 0.2	99.84 ± 0.2
(Ge-Nco)	6.24 ± 0.2	49.8 ± 0.2	12.48 ± 0.2	49.48 ± 0.2	12.48 ± 0.2	49.48 ± 0.2

## Data Availability

The datasets used and/or analysed during the current study are available from the corresponding author on reasonable request.
